# Refined purification strategy for reliable proteomic profiling of HDL_2/3_: Impact on proteomic complexity

**DOI:** 10.1038/srep38533

**Published:** 2016-12-05

**Authors:** Michael Holzer, Sabine Kern, Ruth Birner-Grünberger, Sanja Curcic, Akos Heinemann, Gunther Marsche

**Affiliations:** 1 Institute of Experimental and Clinical Pharmacology, Medical University of Graz, Austria; 2 Institute of Pathology and Proteomics Core Facility, Center for Medical Research, Medical University of Graz, Austria

## Abstract

Proteomics have extended the list of high-density lipoprotein (HDL) associated proteins to about 90. One of the major issues of global protein characterization is establishing specificity of association as opposed to contamination, a fact which has never been addressed for isolated HDL. We have developed a refined purification strategy to isolate HDL by density, followed by purification by size to generate “highly purified” fractions of HDL_2/3_, which allow the reliable quantification of the HDL proteome for biomarker discovery. Mass spectrometry analysis revealed that the proteome of HDL_2/3_ is composed of 10–16 different proteins, which is in striking contrast to previous reports. Importantly, proteomic analysis revealed that many proteins which have recently been described to be associated with HDL, including α-1-antitrypsin, α-2-HS-glycoprotein, serotransferrin, apolipoprotein A-IV and others, are not associated with HDL_2/3_ and are exclusively found in a different molecular weight fraction containing human serum albumin, lipid-poor apolipoprotein A-I and other proteins. Interestingly, proteins found in this lower molecular weight fraction commonly share lipid-binding properties and enrichment of serum with free fatty acids/lysophophatidylcholine led to a significant increase in co-isolation of lipid-binding proteins such as albumin and α-1-antitrypsin. We propose that this refined method might become a standard in proteomic assessment of HDL_2/3_ making data from clinical cohorts more comparable and reproducible.

Cardiovascular disease remains the leading cause of death worldwide indicating the need for suitable predictive disease biomarkers. It is hoped that lipoprotein-specific biomarkers can indicate an individual’s susceptibility to developing disease or to detect the early stages of disease. Recent advances in proteomics have extended the list of HDL-associated proteins to over 90^1^,^2^,^3^,^4^,^5^,^6^,^7^,^8^,^9^,^10^,^11^, suggesting that the composition of HDL is more complex than previously anticipated. Despite the rather low abundance of several newly identified proteins, many have been proposed as biologically active. Proteomic studies identified HDL as being rich in proteins involved in the acute-phase response, complement activation, proteolysis, immunity and many other metabolic pathways^12^. Several patient cohorts, including coronary artery disease, end-stage renal disease, psoriasis and arthritis have been studied using proteomic techniques^13^,^14^ leading to the hypothesis that during chronic disease a specific remodeling of the HDL proteome occurs^15^. It is hoped that these studies will lead to the discovery of lipoprotein-specific biomarkers, which may have the power to indicate an individual’s susceptibility to developing disease or to detect the early stages of disease.

The majority of proteomic studies used HDL isolated through density gradient ultracentrifugation^1^,^2^,^3^,^4^,^6^,^7^. However, the surprisingly high number of HDL-associated proteins raises concerns about the specificity and selectivity of the methodology used. To date the impact of isolation and purification strategies on proteomic diversity of HDL has not been tested yet. Structural analysis of HDL has shown that more than 75% of the lipoprotein surface is covered with apoA-I and A-II^16^, leaving little space for further protein incorporation. To assess whether proteins are truly associated with mature HDL (HDL_2/3_), we developed a purification strategy to isolate “highly purified” fractions of HDL_2/3_ to provide a reliable and accurate analysis of the HDL proteome for biomarker discovery.

## Results

### Molecular characterization of “highly purified” fractions of HDL

**Figure 1:**
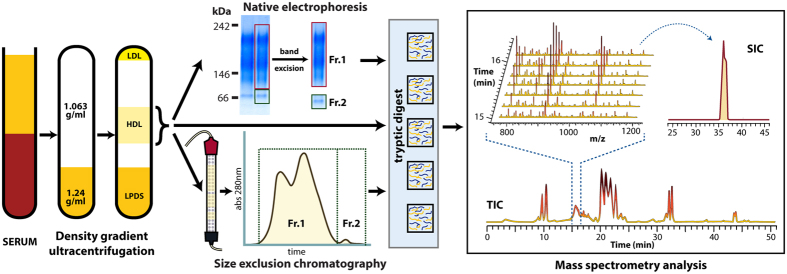
Workflow for isolation of purified HDL and subsequent proteomic profiling . HDL was isolated from pooled serum of healthy controls by two-step density gradient ultracentrifugation. Isolated HDL was either directly used for proteomic assessment or further purified either by size exclusion chromatography or native gel electrophoresis. Bands were excised from native gels corresponding to the molecular size of HDL_2/3_ (100–240 kDa, Fraction 1 (Fr. 1)) and lipid-poor pre-β HDL (50–100 kDa, Fraction 2 (Fr. 2) or fractions were collected after size exclusion chromatography according to the protein trace shown above. The collected fraction were subjected to tryptic digestion and resulting peptide solutions were used for proteomic analysis by LC-MS/MS. Mass spectrometry analysis was done by generating a total ion chromatogram (TIC) and extracting selected ion chromatograms (SIC), which was normalized to the sum of the areas of the whole LC-MS/MS run.

We established a refined strategy to isolate “highly purified” HDL_2/3_ for proteomic characterization. In the first step, instead of using the conventional sequential ultracentrifugation method with very long centrifugation times, we used a previously described one-step density gradient ultracentrifugation method^17^ (Supplemental Fig. 1). To further improve separation, we used longer centrifugation tubes (76 mm), which allowed us for the complete removal of all apoB-containing lipoproteins within one ultracentrifugation step. Complete removal of apoB-containing lipoproteins is a general problem when utilizing the conventional sequential ultracentrifugation approach (Supplemental Fig. 1). In the second step, HDL isolated by ultracentrifugation was further purified by size using either native gel electrophoresis or size exclusion chromatography (Fig. 1). This methodology has the advantage that contaminants that overlap in density can be removed by separation in size. After native gel electrophoresis, bands were excised corresponding to the molecular weight of HDL_2/3_ and pre-β HDL as depicted in Fig. 1.

For samples purified by size exclusion chromatography, we monitored the elution of protein over time and collected fractions corresponding to mature HDL_2/3_ and pre-β HDL (Fig. 1). The excised bands or protein fractions were subjected to tryptic digestion and resulting peptide solutions were used for proteomic analysis by LC-MS/MS. For quantification, the precursor ion area of each detected protein was calculated from the selected ion chromatogram (SIC) extracted from the total ion chromatogram (TIC) and normalized to the sum of the areas of the whole LC-MS/MS run. The resulting values correspond to the relative amount of a protein in one sample as compared to its amount in the other samples of the same experiment. This approach is superior to spectral counting since it is more sensitive and has a wider dynamic range by avoiding saturation of high abundance proteins due to dynamic exclusion of detected proteins. Moreover, it is more robust because multiple peptides are quantified per protein instead of simply counting the number of MS/MS spectra matched to a protein.

Our analysis indicated that HDL isolated by ultracentrifugation without additional purification contained 26 different proteins (Table 1). This rather low number of identified proteins is a result of our improved isolation procedure by using a one-step ultracentrifugation in combination with longer centrifugation tubes (Supplemental Fig. 1). The mass spectrometer system and the criteria used for peptide identification were comparable to previous studies investigating HDL (Supplemental Table 1). All identified proteins have been found in prior proteomic studies of HDL. Upon purification of isolated HDL with native gel electrophoresis, the number of HDL_2/3_ associated proteins decreased to 10 (Table 1). As expected, the main constituents were apoA-I and apoA-II, accounting together for over 90% of the total protein mass. The fraction corresponding to the molecular weight of pre-β HDL contained 13 proteins, the main components were apoA-I (42.3%), human serum albumin (HSA) (21.0%) and paraoxonase (13.8%). The same profile was observed when isolated HDL was purified by size exclusion chromatography with apoA-I and apoA-II, accounting together for ~90% of the protein mass (Supplemental Table 2). The HDL_2/3_ fraction contained 16 proteins, while the pre-β HDL containing fraction contained 22 proteins. Notable, the overall number of identified proteins was higher in samples purified by size exclusion chromatography. Importantly, the proteomic analysis revealed that many proteins which have recently been described to be associated with HDL_2/3_, including α-1-antitrypsin, α-2-HS-glycoprotein, serotransferrin, apoA-IV are exclusively associated with the fraction containing lipid-poor apoA-I, HSA and potentially other contaminants (Table 1).

To study the proteome of HDL subtypes in more detail, we excised bands corresponding to HDL_2_ (190–240 kDa) and HDL_3_ (110–190 kDa) from native gels after electrophoresis. Our analysis indicates that HDL_2_ and HDL_3_ were composed of 13 and 10 proteins, respectively (Table 1). Distinct differences between the two HDL subtypes were found, with HDL_2_ having a higher content of apoA-I and apoE, while HDL_3_ was richer in apoA-II and paraoxonase 1 (Table 1). This result is in good agreement with previous reports^2^ and underlines the validity of our approach to investigate the protein composition of HDL subtypes.

### Immunoblotting and cross-linking experiments confirm the distinct separation of proteins between HDL_2/3_ and the pre-β HDL containing fraction

**Figure 2:**
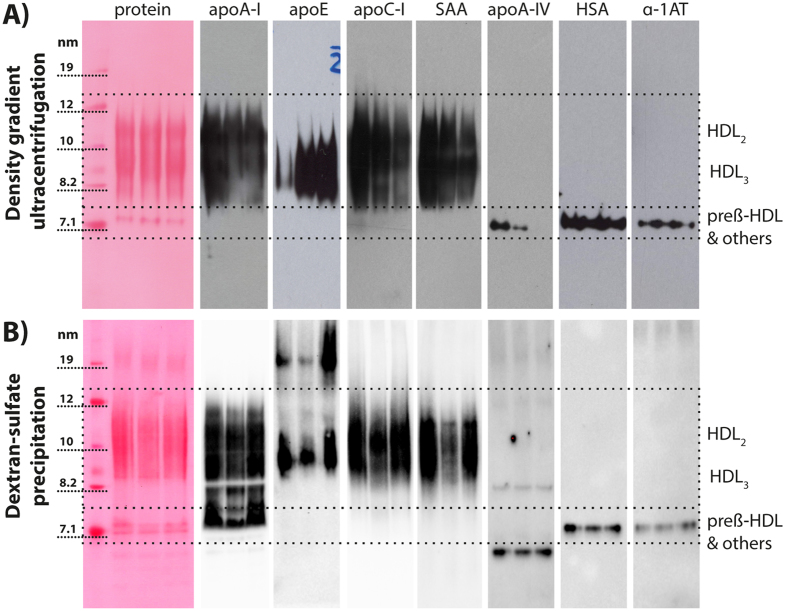
Immunoblot detection of proteins in HDL isolates. HDL was isolated from 3 individual healthy donors by (**A**) two-step density gradient ultracentrifugation or (**B**) dextrane-sulfate precipitation as described in methods. HDL was probed for proteins which were according to mass spectrometry results (Table 1) exclusively associated either with the HDL_2/3_ fraction (apoA-I, apoE, apoC-I and SAA) or with the pre-β HDL containing fraction (apoA-IV, HSA and α-1-AT) (Table 1). HDL was separated by native gel electrophoresis, transferred to PDVF membranes and probed using specific antibodies as described in methods. Total protein was visualized with Panceau Red staining. α-1-AT, α-1-antitrypsin; apo, apoprotein; HSA, human serum albumin.

To further test the hypothesis that proteins in the pre-β fraction are not associated with HDL_2/3_, we performed Western blot analysis of those identified proteins. Based on the result of the proteomic analysis, we chose to probe Western blots of HDL isolated by ultracentrifugation for proteins which were either exclusively associated with the HDL_2/3_ fraction (apoE, apoC-I and SAA), with the pre-β HDL containing fraction (apoA-IV, HSA and α-1-antitrypsin) or in both fractions (apoA-I) (Table 1). The Western blot analysis confirmed mass spectrometry results and showed a distinct pattern between proteins associated with the HDL_2/3_ fraction or with the pre-β HDL fraction (Fig. 2A). Ultracentrifugation exposes HDL to high gravitational forces during isolation, a factor which might influence its protein composition. To isolate HDL without the need for ultracentrifugation, we selectively precipitated HDL with dextran sulfate, a sulfated polyanion which forms insoluble complexes with LDL, HDL and other lipoproteins depending on the concentration used^18^. As expected, HDL isolated by dextran-sulfate precipitation showed more impurities when compared with HDL isolated by ultracentrifugation, which became evident after native gel electrophoresis (Fig. 2B). Western blots from dextran-sulfate isolated HDL indicated a similar distribution of proteins between HDL_2/3_ fraction and pre-β HDL, with HSA and α-1-antitrypsin exclusively found in the lower molecular weight fraction, corresponding to the size of pre-β HDL and other proteins (Fig. 2). However, the distribution of apoA-IV was different between the two isolation methods. While apoA-IV was found only in the pre-β HDL fraction in HDL from ultracentrifugation (molecular weight range from 7–7.7 nm), the majority of apoA-IV in dextrane-sulfate isolated HDL was found at around 45 kDa, corresponding to the molecular weight of a single apoA-IV molecule. A minor fraction of apoA-IV was also found as higher molecular weight aggregate (Fig. 2B).

A recent report has provided initial experimental data on the ability of reconstituted HDL (phospholipid vesicles containing apoA-I) to bind α-1-antitrypsin^19^. However, we were not able to detect α-1-antitrypsin in HDL_2/3_ isolated by ultracentrifugation or dextrane-sulfate precipitation. To further prove that α-1-antitrypsin is not directly HDL_2/3_ associated, we immune-precipitated α-1-antitrypsin from serum to test whether apoA-I, the main component of HDL, co-precipitates. Western blot analysis confirmed the presence of α-1-antitrypsin in isolates, but apoA-I was not detected (Supplemental Fig. 2).

**Figure 3:**
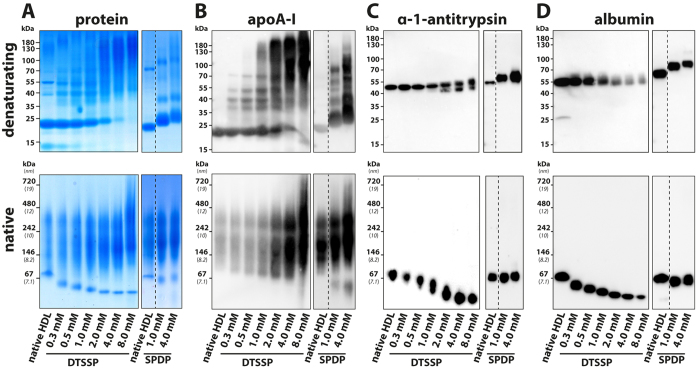
Immunoblot detection of protein in HDL isolates after cross-linking. HDL isolated by density gradient ultracentrifugation was cross-linked prior to electrophoresis with DTSSP (short chain cross-linker, spacer length 12 Å) or with SPDP (PEGylated SPDP, long-chain cross-linker, 54 Å). HDL was separated under denaturating (SDS-Page, upper panels) or non-denaturating (native) conditions (lower panels), stained with Coomassie brilliant blue for total protein (**A**) or transferred to PDVF membranes and probed using specific antibodies for apoA-I (**B**), α-1-antitrypsin (**C**) or human serum albumin (HSA) (**D**). Molecular weight is indicated on the right. The dashed line indicates where gels and blots have been cut to put the appropriate control next to the cross-linked sample from the same gel or blot.

To exclude the possibility that proteins have been displaced from HDL during gel electrophoresis, we cross-linked proteins in HDL preparations prior to gel electrophoresis. If protein interaction occurs, a cross-linker would connect the interacting proteins and cause a molecular shift visible on immunoblots from native gels or SDS PAGE gels. We used two different cross-linking agents, DTSSP and PEG12-SPDP, with different spacer arm lengths, 12 Å and 54 Å respectively. This allowed us to probe for proteins which interact very closely and proteins which interact more loosely. Crosslinking was efficient evident by a molecular shift of apoA-I, which interacts on the surface of HDL particles and is expected to form dimers and trimers upon crosslinking (Fig. 3A,B). The molecular integrity of native HDL particle did not change significantly after crosslinking with both agents (Fig. 3A, lower panel). Importantly, when the corresponding blots from denaturating gels were probed for HSA and α-1-antitrypsin, we observed no shift to a higher molecular weight, suggesting that the probed proteins were not cross-linked to other proteins (Fig. 3C,D upper panel). Furthermore, when blots from native gels were probed for HSA and α-1-antitrypsin, we found that both proteins were exclusively associated with the pre-β HDL containing fraction (Fig. 3C,D lower panel). While equal amounts of proteins have been loaded on all lanes, the apoA-I antibody always showed increased affinity upon crosslinking on native gels (3 B, lower panel). The in-gel migration behavior of α-1-antitrypsin and human serum albumin (HSA) was changed after crosslinking (3 C, D, lower panels), presumably by a change in molecular structure/charge. In summary, these results suggest that HSA and α-1-antitrypsin do not directly interact with other HDL proteins.

### Re-analysis of previous proteomic studies reveals potential sources of contamination

As a comparison to prior investigations, we collected data from recent research papers analyzing the HDL proteome. Seven research papers were chosen which used similar isolation techniques (density gradient ultracentrifugation), mass spectrometry techniques (Shotgun LC-ESI) and provided quantitative raw data of their mass spectrometry analysis. Data of spectral counts from proteomic measurements of healthy subjects were used to calculate a semi-quantitative estimate of the abundance of each identified protein. For example if 100 spectral counts were identified by database search to be related to a specific protein and the overall sum of spectral counts was 2000, we would estimate that the identified protein accounts for about 5% of the total protein (The collected raw data and calculations are given in the Supplemental Tables 3, 4 and 5).

Surprisingly, our analysis revealed that across recent reports only 41% of all spectral counts are related to apoA-I (Table 2). Furthermore, the content of HSA was 7.3% in average (ranged between 0.35–16.35%), and the content of apoB was 5.5% in average (range from 0–19.05%) (Table 2). These estimates are in striking contrast to our analysis of purified HDL_2/3_, the most abundant form of HDL. ApoA-I alone accounted for 70–80% in purified HDL_2/3_ and was completely devoid of HSA and apoB (Table 1). Although, the isolation and quantification methodology differed from our approach, this comparison strengthens our hypothesis that additional purification steps are needed to eliminate impurities from the HDL_2/3_ fraction to allow a correct analysis of the HDL proteome.

### Fatty acid/lysophospholipid enrichment of serum leads to increased co-isolation of lipid-binding proteins

**Figure 4:**
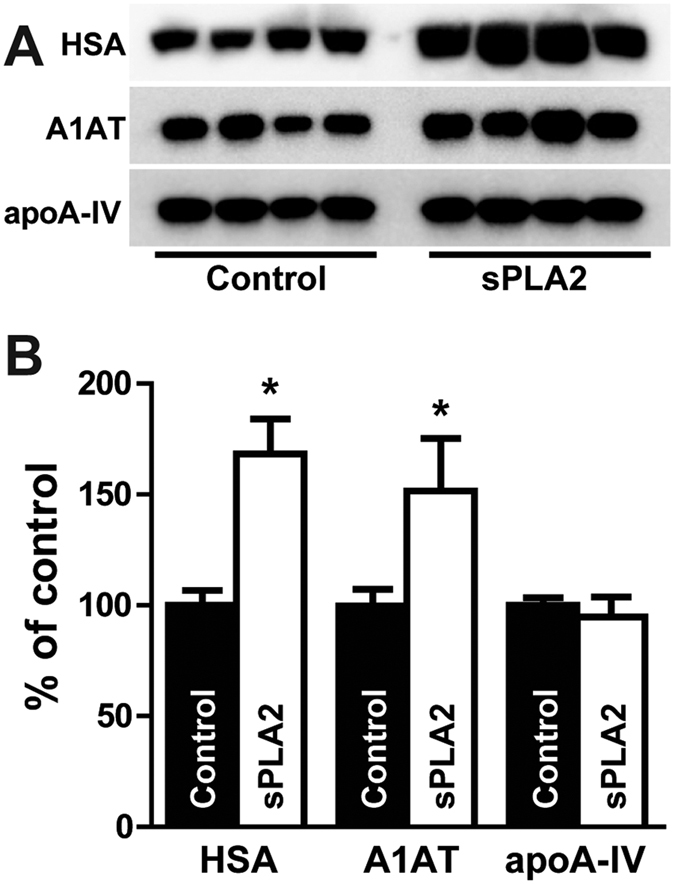
Immunoblot detection of proteins in HDL isolates after sPLA_2_ treatment. Serum was treated with 0.2 μg/ml sPLA_2_ overnight to increase the content of free fatty acids and lysophosphatidylcholine. Afterwards, HDL was isolated by density gradient ultracentrifugation and the protein composition of the preparation was assessed by Western blot. (**A**) HDL preparations were separated by SDS-Page, blotted and probed using specific antibodies for apoA-IV, α-1-antitrypsin (A1AT) or human serum albumin (HSA). Shown is a representative example of one preparation run in quadruplicate. (**B**) Blots were imaged on a Chemidoc Imaging machine and analyzed with ImageLab 5.2. Results represent the combined analysis of 3 independent HDL preparations run in quadruplicates on SDS Page gels. *p < 0.05 vs. control.

Our data suggest that especially proteins with lipid-binding abilities, such as HSA, α-1-antitrypsin or apoA-IV are prone to be contaminants of HDL isolates. To test this hypothesis, we treated serum prior to HDL isolation with secretory phospholipase A_2_ (sPLA_2_) to increase the content of lysophospatidylcholine and free fatty acids. Upon centrifugation and isolation of HDL, we assessed the amount of co-isolated HSA, α-1-antitrypsin and apoA-IV. As seen in Fig. 4, we found that HDL isolates from sPLA2-treated serum contained about 1.7-times more HSA and 1.4-times more α-1-antitrypsin than control serum, while the content of apoA-IV was unchanged likely because apoA-IV binds intact phospholipids. This result suggests that enrichment in free fatty acids in serum can lead to a higher amount of contaminating proteins presumably by enhanced lipid binding and reduction in particle density.

## Discussion

In this study, we have developed a purification strategy to isolate “highly purified” fractions of HDL_2/3_ which allow the accurate and reliable quantification of the HDL_2/3_ proteome by mass spectrometry. This is of particular importance since proteomic profiling of HDL is used in clinical studies to identify disease related biomarkers.

Our data clearly indicate that the proteome of HDL_2/3_ is not as complex as previously anticipated and is composed of 10–16 proteins. This is in striking contrast to previous reports claiming that the HDL proteome is comprised of up to 95 proteins. Our analysis revealed that the majority of potential HDL associated proteins that have been newly discovered by proteomic analysis are not associated with mature forms of HDL and are exclusively found in the fraction containing lipid-poor apoA-I, HSA and other proteins. This observation generally raises the question whether several novel HDL associated proteins identified by proteomics in recent years are truly HDL associated or just contaminants of insufficient purification of isolated HDL in the various isolation strategies used.

The majority of proteomic studies isolated HDL by ultracentrifugation, a method which utilizes the difference in density between lipoproteins and other proteins. It is innate to this method that a complete separation cannot easily be achieved, since unrelated proteins, especially proteins with lipid-binding abilities can have similar densities. Proteins which are frequently seen in the density range of HDL (1.063–1.210 g/ml) are HSA, LDL and Lp(a). This is confirmed by our review of recent papers that describe the HDL proteome, highlighting the problem that HDL fractions are prone to be contaminated with other serum proteins. In these studies, high contents of HSA (mean = 7.3%; range: 0.35–16.35%), as well as apoB (mean = 5.5%; range: 0.00–19.05%), the major apoprotein of LDL and Lp(a) were observed. LDL has an only slightly lower density than HDL (HDL: 1.063–1.210 g/ml vs. LDL: 1.019–1.063 g/ml) and is a potential source of impurities in HDL isolates. The high contents of apoB, the major apoprotein of LDL in recent HDL proteome papers raise the question whether LDL contaminations generally impact proteomic characterization of HDL, since LDL also carries proteins such as apoC-II, apoC-III, apoE and Lp-PLA2^20^,^21^. Furthermore, the analysis of the HDL “lipidome” might be profoundly affected, given that LDL particles are mainly composed of lipids.

HSA is a well-studied plasma “cargo” protein that binds a variety of endogenous compounds, including proteins and lipids as well as exogenous compounds such as drugs^22^,^23^. Importantly, proteins which have been associated with HDL since the dawn of proteomics era can all be found in isolated HSA fractions, including apoA-VI, α-1-antitrypsin, retinol-binding protein 4 and complement C3^24^,^25^,^26^, raising the possibility that these proteins are rather co-isolates like HSA than truly HDL associated.

Several studies have confirmed the presence of α-1-antitrypsin in HDL isolated by ultracentrifugation^27^ or size-exclusion chromatography^19^. However, a recent study has provided data that *in vitro* reconstituted nascent HDL is able to bind α-1-antitrypsin^19^. In contrast, our data suggest that HDL isolated by ultracentrifugation contains a small amount of α-1-antitrypsin, but upon purification into HDL_2/3_- and pre-β HDL-containing fraction, α-1-antitrypsin could only be found in the fraction containing pre-β HDL, HSA and other proteins. To exclude the possibility that the high gravitational force present during ultracentrifugation leads to shedding of proteins from HDL, we isolated HDL by dextrane-sulfate precipitation. Upon immunoblot analysis, dextrane-sulfate precipitated HDL and HDL isolated by ultracentrifugation showed a similar protein distribution, strengthening our hypothesis that several proteins (Fig. 2), including α-1-antitrypsin and HSA are exclusively located in the fraction containing pre-β HDL and other proteins. In addition, we performed co-immunoprecipitation experiments with specific α-1-antitrypsin antibodies, to see whether apoA-I, the main HDL component, would be co-isolated. While α-1-antitrypsin was clearly precipitated, we were not able to detect apoA-I (Supplemental Fig. 2). Since the additional purification steps performed in our study might have led to the removal of low-affinity associated proteins from HDL, we cross-linked proteins in HDL preparations prior to gel electrophoresis purification. While crosslinking was efficient (evident by the formation of dimers and trimers of apoA-I) there was no indication that proteins found in the pre-β HDL containing fractions were associated with HDL_2/3_ before purification. We specifically tested HSA or α-1-antitrypsin, making it unlikely (i) that these proteins have been displaced during the electrophoresis step or (ii) are likely HDL_2/3_ associated proteins.

In addition, crosslinking of HDL-apolipoproteins before native gel electrophoresis did not provide evidence that α-1-antitrypsin directly binds to any other HDL protein since no molecular shift of α-1-antitrypsin was visible on immunoblots from both, native and denaturating gels. Our data suggest that these proteins likely interact with lipids, generating particles with a similar density of lipid-poor apoA-I/pre-ß HDL. Of note, HSA is able to bind up to seven equivalents of fatty acids at multiple binding sites^23^. This would agree with our previous observation that the apparent HDL content of HSA strongly correlated with apoA-VI, α-1-antitrypsin, transthyretin, and retinol-binding protein 4 in isolated HDL^3^. In line with that notion, a recent study demonstrated that α-1-antitrypsin combines with plasma fatty acids, altering enzyme activities and properties^28^. We tested this hypothesis by treating serum with sPLA_2_ to increase the serum levels of free fatty acids and lysophosphatidylcholine, which lead to significant higher contents of HSA and α-1-antitrypsin in isolated HDL. These experiments suggest that free fatty acid binding by presumably a variety of proteins might cause them to co-isolate with HDL. This could be of particular relevance in patients with chronic inflammatory diseases, such as coronary artery disease, rheumatoid arthritis or chronic kidney disease, where higher activities of sPLA_2_ have been reported^29^,^30^,^31^,^32^, thereby significantly bias the proteomic assessment of isolated HDL.

Overall, the presented method is fast and yields a well-defined HDL_2/3_ fraction with high purity, by removing potential contaminants. Given that the presented method is rapid and simple, we hope that this method might become a standard in proteomic assessment for HDL_2/3,_ making data from different laboratories more comparable and reproducible.

## Online Methods

### Blood collection

Blood was sampled from healthy control subjects after all subjects signed an informed consent form in agreement with the Institutional Review Board of the Medical University of Graz. All methods were carried out in accordance with the approved guidelines. The study protocol and all study procedures were reviewed and approved by the ethics committee of the Medical University of Graz, Austria (Vote Nr.: 21–523 ex 09/10).

### Isolation of HDL

HDL was isolated from serum either by a two-step density gradient ultracentrifugation method^17^ or via dextran sulfate precipitation. For the two-step density gradient ultracentrifugation method, serum density was adjusted with potassium bromide (Sigma, Vienna, Austria) to 1.24 g/ml and a two-step density gradient was generated in centrifuge tubes (16 × 76 mm, Beckman) by layering the density-adjusted plasma (1.24 g/ml) underneath a NaCl-density solution (1.006 g/ml) as described^17^. Tubes were sealed and centrifuged at 65.000 rpm (415.000 g) for 6 hours in a 90Ti fixed angle rotor (Beckman Instruments, Krefeld, Germany). After centrifugation, the HDL-containing band was collected, desalted via PD10 columns (GE Healthcare, Vienna, Austria) and immediately used for experiments.

For HDL isolation from serum by dextrane sulfate, we used a commercial available kit from Cell Biolabs (Nr.: STA-607). The isolation was performed according to the manufacturer’s instructions.

### Size exclusion chromatography

In brief, a NGC QUEST FPLC System (Bio-Rad) equipped with a Superdex 200 Increase 10/300 column (GE Healthcare Europe GmbH, Munich, Germany) was used with 10 mM Tris, 150 mM NaCl, pH 7.4 as running buffer. After loading, HDL samples were separated with a constant flow of 0.5 ml/min, and fractionation was started after 18 min with 0.25 ml per fraction.

### Cross-linking of proteins

DTSSP (3,3′-dithiobis(sulfosuccinimidyl propionate)), ThermoFisher, Vienna, Austria) cross-linker was dissolved in aqua dest. to 25 mM. 100 μl of isolated HDL (6 mg/ml in 0.1 M phosphate puffer, 0.15 M NaCl; pH 7.2) were incubated with 0.25–8 mM DTSSP for 30 min at room temperature. The concentration range used represents a 3–100-fold molecular excess of the cross-linker. The reaction was stopped by adding 20 μl 1 M Tris, pH 7.4 and incubation for 15 min. Afterwards, samples were de-salted and immediately used for experiments.

PEG12-SPDP (PEGylated, long-chain SPDP crosslinker, ThermoFisher, Vienna, Austria) was dissolved in DMSO to 20 mM. 100 μl of isolated HDL (6 mg/ml in in 0.1 M phosphate puffer, 0.15 M NaCl; pH 7.2) were incubated with 1 or 4 mM PEG12-SPDP for 30 min at room temperature, representing a 12.5 or 50-fold molecular excess of the cross-linker. To stop the reaction, samples were de-salted via a PD-10 column to remove un-bound cross-linker.

### Immunoprecipitation of α-1-antitrypsin

Antibody coupling and immunoprecipation was performed with a commercial available kit (Dynabeads Co-IP Kit, Nr.:14321D, Life Technologies) according to the manufacturers instructions. Briefly, a rabbit monoclonal antibody (Clone number: EPR9090–71, Abcam) was covalently coupled to M-270 epoxy magnetic beads, in a ratio of 5 μg antibody per mg beads. Antibody-beads conjugates wer incubated with serum at 4 °C for 30 min. After washing, the bound proteins were eluted and immediately used for immunoblots detection of proteins. To confirm that the detection was valid, positives controls for α-1-antitrypsin (SIGMA) and apoA-I (courtesy of Wadsack C.) were tested in parallel.

### Gel electrophoresis and blotting

For native gel electrophoresis, isolated HDL (5–30 μg protein per lane) was separated by gradient gel electrophoresis (4–16% NativePage; Life Technologies, Vienna, Austria) under nonreducing and nondenaturing conditions. Afterwards, gels were either stained with a freshly prepared solution of Coomassie Brilliant Blue G-250 overnight (Thermo Scientific, Rockford, USA) or used for blotting. A high molecular weight marker (NativeMark, Life Technologies, Vienna, Austria), containing bovine serum albumin (7.1 nm), lactate dehydrogenase (8.2 nm), B-phycoerythrin (10.5 nm, apoferritin band 1 (12.2 nm) and apoferritin band 2 (18.0 nm) was used to estimate the size of HDL. For proteomic analysis, bands were excised after electrophoresis as shown in Fig. 1. For denaturating gel electrophoresis, isolated HDL (5–30 μg protein per lane) was separated by SDS polyacrylamide gel electrophoresis on (Novex 4–20% gels, Life Technologies, Vienna, Austria) under reducing and denaturating conditions. Gels were run at constant voltage of 150 V for 120 min, in SDS running buffer containing 25 mM Tris, 50 mM Glycine and 3.5 mM SDS. A molecular weight marker was used for size determination (Page ruler, Thermo Fisher).

For blotting, gels were transferred to polyvinylidene difluoride membranes with 100 V for 60 or 90 min at 4 °C for native gels or SDS Page respectively. Membranes were probed with the following primary antibodies diluted in 5% milk overnight at 4 °C: apoA-I, (Nr.: NB100654491, 1:10000 dilution, Novus Biologicals), apoA-I, (Nr.: ab52945, Abcam), apoC-I (Nr.: BP2081, 1:1000 dilution, Acris), SAA (Nr.: RAS-H-SAA-A8, 1:1000 dilution, courtesy of G. Kostner, Graz, Austria), apoA-IV (Nr.: K381, 1:10000 dilution, courtesy of G. Kostner), HSA (Nr.: ab83465, 1:1000 dilution, Abcam, Cambridge, UK), α-1-antitrypsin (Nr.: ab179443, 1:1000 dilution, Abcam, Cambridge, UK). ApoA-I was tested with two different antibodies, since the antibody from NOVUS we initially used (Nr.: NB100–654491) is no longer available. Therefore, we switched to an apoA-I antibody from Abcam (Nr.: ab52945) for the experiments shown in Fig. 2B and Supplemental Fig. 2. Membranes were washed and incubated with secondary HRP-conjugated antibodies for 2 h at ambient temperature. Membranes were carefully washed and developed using a reagents and detection was performed on a Chemidoc Touch imaging system (Bio-Rad, Vienna, Austria).

### LC-MS/MS analysis

In total 50 μg of purified HDL were precipitated with acetone, dissolved in 100 mM ammoniumhydrogencarbonate buffer, reduced by adding 5 mM dithiothreitol and alkylated using 20 mM iodoacetamide before the samples were digested with Promega modified trypsin according to the manufacturer’s protocol. For LC-MS/MS measurement 250 ng aliquots were acidified to 0.1% formic acid and injected. The protein bands were excised from the native gel and reduced, alkylated and digested with Promega modified trypsin according to the method of Shevchenko *et al*. (1996) [Anal. Chem.1996, 68, 850–858]. Peptide extracts were dissolved in 0.3% formic acid and 5% acetonitrile. One fifth of the total protein amount was used for the separation by nano-HPLC (Dionex Ultimate 3000) equipped with a C18, 5 μm, 100 Å, 500 μm × 0.3 mm enrichment column and an Acclaim PepMap RSLC nanocolumn (C18, 2 μm, 100 Å, 50 cm × 0.075 mm) (all Thermo Fisher Scientific, Vienna, Austria). Samples were concentrated on the enrichment column for 2 min at a flow rate of 20 μl/min with 0.1% formic acid as isocratic solvent. Separation was carried out on the nanocolumn at a flow rate of 200 nl/min using the following gradient, where solvent A is 0.1% formic acid in water and solvent B is a mixture of 80% acetonitrile in water containing 0.1% formic acid: 0–2 min: 4% B; 2–180 min: 4–28% B; 180–255 min: 28–50% B, 255–260 min: 50–95% B, 260–279 min: 95% B, 280–300 min: re-equilibration at 4% B. The sample was ionized in the nanospray source equipped with stainless steel emitters (ES528, Thermo Fisher Scientific, Vienna, Austria) and analyzed in a Thermo LTQ FT-Ultra mass spectrometer (Thermo Fisher Scientific, Waltham, MA, USA) in positive ion mode by alternating full scan MS (m/z 300 to 2000, resolution 50.000) in the ion cyclotron and MS/MS by collision induced dissociation of the 3 most intense peaks in the ion trap with dynamic exclusion enabled. Proteome Discoverer 1.4 (Thermo Fisher Scientific, Waltham, MA, USA) and Mascot 2.4 (MatrixScience, London, UK) were used for MS/MS data analysis by searching the human SwissProt public database downloaded on June 16th 2015. Detailed search criteria: enzyme: trypsin, maximum missed cleavage sites: 2, carbamidomethylation of cysteine residues was set as fixed modification, oxidation on methionine as variable modification, precursor mass tolerance +/−10 ppm, product mass tolerance +/−0.8 Da. Data were filtered according to stringent peptide acceptance criteria: Peptide Confidence: high, Max. Peptide rank: 1, Peptide mass deviation: 10 ppm, Min. Number of peptides: 2, count only rank 1 peptides, Mascot Ion Score of at least 20 and rank 1 in Mascot search, and min. 2 peptides per protein. Additionally the precursor ion areas were calculated and matched to each protein with Proteome Discoverer 1.4. Areas of each protein were normalized to the sum of the areas of the whole LC-MS/MS run, respectively. Raw data from proteomic measurements can be found in the Supplement (Supplemental Table 6a and b).

## Additional Information

**How to cite this article**: Holzer, M. *et al*. Refined purification strategy for reliable proteomic profiling of HDL_2/3_: Impact on proteomic complexity. *Sci. Rep.* **6**, 38533; doi: 10.1038/srep38533 (2016).

**Publisher's note:** Springer Nature remains neutral with regard to jurisdictional claims in published maps and institutional affiliations.

## Supplementary Material

Supplementary Information

## Figures and Tables

**Table 1:**
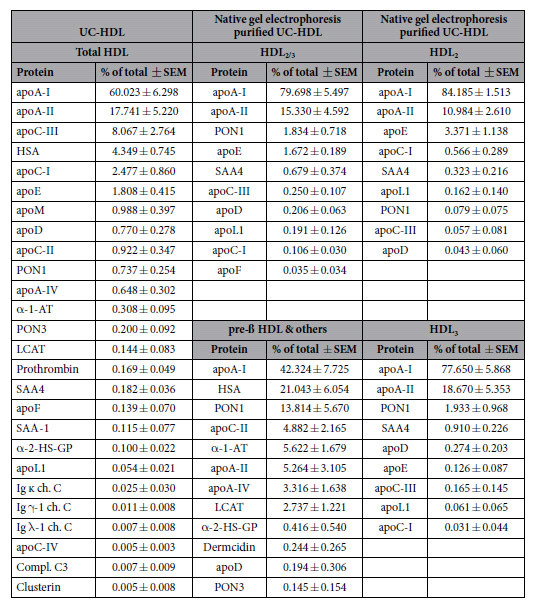
Proteomic analysis of isolated and purified HDL-containing fractions.

Results represent 3 independent measurements in duplicate of isolated HDL from pooled human sera. List represents all proteins detected in at least two out of six samples per group. The complete list can be found in Supplemental Table 5. apo. apoprotein; AT. Antitrypsin; ch. chain; Compl. Complement; GP. Glycoprotein; Ig. Immunoglobulin; LCAT. lecithin cholesterylester transfer protein; PON. Paraoxonase; SAA. Serum amyloid A; TF. transferrin; UC. Ultracentrifugation.

**Table 2:** Semi-quantitative estimates of protein abundance based on recently published research papers describing the HDL proteome.

Protein name	% of total spectral counts							
	Vaisar *2007. JCI*^*1*^	Davidson *2009. ATVB*^*2*^	Holzer *2011. JASN*^*3*^	Holzer *2012. JLR*^*4*^	Weichhart *2012. JASN*^*5*^	Sreckovic *2013. BBA*^*6*^	Riwanto *2013. Circ*^*7*^	Average (%)
apoA-I	23.886	37.515	60.066	59.319	23.555	55.449	27.853	**41.09**
apoA-II	8.064	8.357	10.434	4.883	4.711	13.590	2.337	**7.48**
HSA	16.246	0.349	1.948	8.265	13.945	8.298	2.120	**7.31**
apoE	4.244	8.423	6.494	3.063	6.972	2.268	7.418	**5.55**
apoB	1.368	13.840	—	1.877	0.817	1.475	19.049	**5.49**
apoC-III	2.476	1.927	4.835	4.399	2.701	5.384	1.359	**3.30**
SAA4	2.924	5.416	5.470	2.192	3.078	1.106	2.799	**3.28**
apoC-I	1.745	4.120	4.358	3.566	2.387	2.831	1.576	**2.94**
apoD	1.651	3.638	2.338	1.357	4.711	0.811	4.185	**2.67**
apoA-IV	3.278	0.150	—	0.609	7.852	—	3.071	**2.14**
apoC-II	2.382	1.927	1.227	0.599	0.628	5.384	1.033	**1.88**
PON 1	2.806	0.366	0.058	2.333	3.894	0.738	2.609	**1.83**
apoM	1.745	4.652	1.703	0.826	2.010	0.175	1.658	**1.82**
apoL1	1.485	3.555	0.029	0.830	2.136	0.535	3.016	**1.66**
α-1-AT	4.504	0.199	—	0.529	—	0.747	2.092	**1.15**
SAΑ 1/2	2.169	1.229	0.808	0.565	1.570	0.065	0.625	**1.00**
apo(a)	—	0.781	0.058	0.253	0.565	—	2.446	**0.59**
apoF	0.943	1.545	—	0.263	0.691	—	0.380	**0.55**
Clusterin	1.132	0.233	—	0.084	1.382	—	0.761	**0.51**
HRP	0.920	0.216	—	—	1.822	—	0.217	**0.45**
Transthyretin	1.674	0.050	—	0.222	1.193	—	—	**0.45**
Compl. C3	0.424	—	—	0.441	0.314	—	1.848	**0.43**
PLTP	0.660	0.498	—	—	0.628	—	1.060	**0.41**
apoH	1.934	—	—	—	0.188	0.535	0.109	**0.40**
PON 3	0.731	0.282	—	—	0.879	—	0.679	**0.37**
α-2-HS-GP	1.391	—	—	0.148	0.565	0.065	0.190	**0.34**
PC oxidase	0.802	—	—	—	0.188	—	1.250	**0.32**
LCAT	1.438	—	—	—	0.503	—	0.299	**0.32**
TF	0.236	—	—	0.262	1.445	0.065	0.109	**0.30**
Vitamin D BP	1.533	—	—	0.062	0.440	0.009	—	**0.29**
Haptoglobin	—	—	—	0.113	1.570	—	0.109	**0.26**
Vitronectin	0.990	—	—	—	0.565	—	0.054	**0.23**

Raw data (spectral counts) from healthy control groups were collected from the indicated published studies and used to estimate protein abundance. Shown are proteins with an abundance of more than 0.2%. The complete list of proteins and the calculation can be found in Supplemental Tables 2, 3 and 4. “−”, not detected; apo, apoprotein; AT, antitrypsin; BP, binding protein; Compl., complement; GP, glycoprotein; HSA, human serum albumin; HRP, haptoglobin related protein; LCAT, lecithin cholesterylester transfer protein; PLTP, phospholipid transfer protein; PON, paraoxonase; SAA, serum amyloid A; TF, transferrin; UC, ultracentrifugation.
