# Supplementary material for: Refined purification strategy for reliable proteomic profiling of HDL2/3: Impact on proteomic complexity
Source: Sci Rep. 2016 Dec 5;6:38533. doi: 10.1038/srep38533 (PMC5137140; doi:10.1038/srep38533)
Supplement: Supplementary Information [file srep38533-s1.pdf]

Refined purification strategy for reliable proteomic profiling of $HDL_{2/3}$: Impact on proteomic complexity

Authors: [1]Michael Holzer, PhD*; [1]Sabine Kern, TA [2]; Ruth Birner-Grünberger, PhD; [1]Sanja Curcic, PhD; [1]Akos Heinemann, MD; and [1]Gunther Marsche, PhD.

Supplemental Figures:

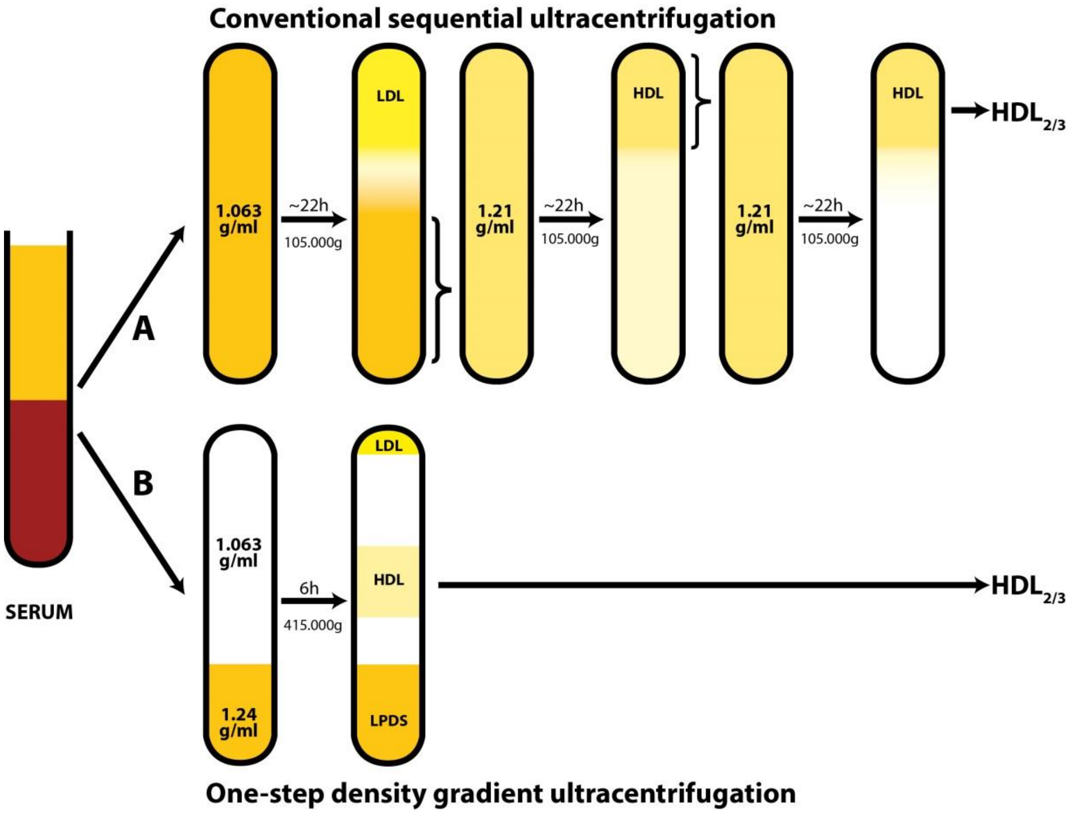

Supplemental Figure 1: Comparison of methods used to isolate HDL by ultracentrifugation. A) For sequential ultracentrifugation, serum is adjusted to 1.063 g/ml with potassium bromide (KBr) and centrifuged for 22 hours at 105.000 g. This step concentrates lipoproteins with densities below 1.063 g/ml in the top layer of the tube, being chylomicrons, very low-density lipoprotein and low-density lipoprotein. The top layer can be removed by slicing the tube. The remaining fraction will be adjusted to a density of 1.21 g/ml with KBr. After another 22 hours of centrifugation, the HDL containing fraction is concentrated in the top layer and can be recovered by slicing the tube. Complete removal of apoB-containing lipoproteins is a general problem when utilizing the conventional sequential ultracentrifugation approach, since the interphase between HDL, LDL and the remaining plasma proteins is difficult to find and therefore often requires additional purification steps. Therefore, the density of the recovered HDL fraction is adjusted again to 1.21 g/ml and centrifuged for 22 hours. **B)** For one step density gradient ultracentrifugation, serum is adjusted to 1.24 g/ml with KBr. The serum fraction is overlaid with PBS adjusted to 1.063 g/ml with PBS and the tube sealed. After centrifugation for 6 hours at 415.000 g, the HDL containing fraction can be found in the middle layer, while chylomicrons, very low-density lipoprotein and low-density lipoprotein can be found in the top layer. HDL can be recovered from the tube by punctuation with a needle and a syringe.

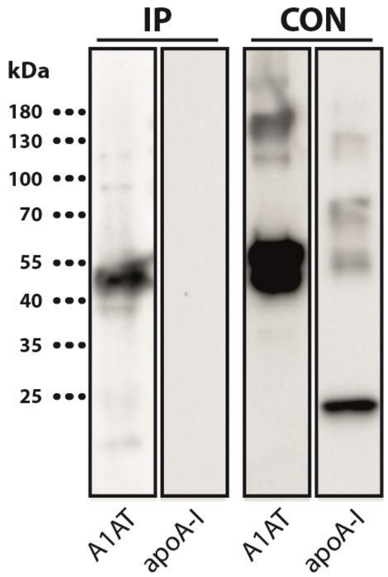

Supplemental Figure 2: Immunoprecipitation of α-1-antitrypsin. Antibodies specific against α-1-antitrypsin were covalently coupled to magnetic beads. Antibody-beads conjugates were incubated with serum for 30 min to allow binding of α-1-antitrypsin. After immunoprecipitation, the isolate was separated on SDS-Page, blotted and tested with antibodies against α-1-antitrypsin (A1AT) and apoA-I (lane 1&2, header: IP). Positives controls for α-1-antitrypsin (0.1µg) and apoA-I (0.1µg) were tested in parallel with the same antibodies (lane 3&4, header: CON).

Supplemental Tables:

Supplemental Table 1: Comparison between mass spectrometry measurements and parameters of analysis within proteomic studies investigating HDL

**Vaisar et al.**<br><br>**2007, JCI[1]**	LCQ Deca ProteomeX ion trap mass spectrometer (Thermo Electron). MS/MS spectra were searched against the human International Protein Index (IPI) database v.3.01, using the SEQUEST search engine (Thermo Electron)	• up to 1 missed cleavage site<br>• at least 2 unique peptides
**Davidson et al.**<br><br>**2009, ATVB[2]**	Sciex/Applied Biosystems QSTAR XL mass spectrometer. SwissProt human database (update 071807) using 3 different search engines: MASCOT (Matrix Sciences), Phenyx (GenBio, Geneva Switzerland), and X! Tandem (The Global Proteome Machine, www.theGPM.org).	• product mass tolerance = ±0.15 Da<br>• up to 2 missed cleavage sites<br>• at least 2 unique peptides<br>• found with 2 out of 3 search engine algorithm
**Holzer et al.**<br><br>**2011, JASN[3]**	LTQ-FT mass spectrometer (Thermo Fischer Scien.) Human NCBI nonredundant public database searched with Spectrum mill Rev. A.03.03.078 (Agilent) and Mascot 2.2 (MatrixScience, UK).	• precursor mass tolerance = ±0.05 Da<br>• product mass tolerance = ±0.7 Da<br>• up to 2 missed cleavage sites<br>• at least 2 unique peptides
**Holzer et al.**<br><br>**2012, JLR[4]**	LTQ-FT mass spectrometer (Thermo Fischer Scien.) Human SwissProt public database downloaded on May 4, 2011, searched with Spectrum Mill Rev. A.03.03.084 SR4 (Agilent).	• precursor mass tolerance = ±0.05 Da<br>• product mass tolerance = ±0.7 Da<br>• up to 2 missed cleavage sites<br>• at least 2 unique peptides
**Weichhart et al.**<br><br>**2012, JASN[5]**	XCT-Ultra ion trap mass spectrometer (Agilent) Spectrum Mill Proteomics software (Agilent version A.03.03.081), searched against the SwissProt Database for human proteins (version 14.3)	• precursor mass tolerance = ±1.5 Da<br>• product mass tolerance = ±0.7 Da<br>• up to 2 missed cleavage sites<br>• one unique protein with peptide score > 13
**Sreckovic et al.**<br><br>**2013, BBA[6]**	LTQ-FT mass spectrometer (Thermo Fischer Scien.) Human NCBI nonredundant public database (downloaded on March 11th, 2011) searched with Spectrum mill Prot. (Agilent A.03.03.078) and Mascot 2.2 (MatrixScience).	• precursor mass tolerance = ±0.05 Da<br>• product mass tolerance = ±0.7 Da<br>• up to 2 missed cleavage sites<br>• 2 or more identified peptides
**Riwanto et al.**<br><br>**2013,Circulation[7]**	LTQ-Orbitrap mass spectrometer (Thermo Fischer Scien.) Mascot server 2.2 against a human protein database (EBI, release date: 19/01/2010).	• precursor mass tolerance = ±6 ppm<br>• product mass tolerance = ±0.5 Da<br>• up to 1 missed cleavage site<br>• at least 2 unique peptides<br>• present in 4 out of 6 samples
**Holzer et al.**	LTQ FT-Ultra mass spectrometer (Thermo Fisher Scien.) Proteome Discoverer 1.4 (Thermo Fisher Scientific) and Mascot 2.4 (MatrixScience, UK) were used for MS/MS data analysis by searching the human SwissProt public database downloaded on June 16th 2015.	• precursor mass tolerance = ±10 ppm<br>• up to 2 missed cleavage sites<br>• product mass tolerance = ±0.8 Da<br>• at least 2 unique peptides

Supplemental Table 2: Proteomic analysis of UC-HDL purified by size exclusion chromatography

HDL 2/3				pre-ß HDL & others			
**name**	**% of total**	**±**	**SEM**	**name**	**% of total**	**±**	**SEM**
ApoA-I	69.680	±	2.499	apoA-I	50.251	±	10.908
ApoA-II	18.236	±	3.564	HSA	13.627	±	4.447
ApoC-III	2.670	±	0.962	PON1	7.757	±	3.999
ApoE	2.068	±	0.242	apoA-II	5.020	±	2.946
ApoC-I	1.682	±	0.690	apoC-II	4.225	±	1.392
ApoM	1.560	±	0.837	α-1-AT	3.826	±	1.479
PON1	1.015	±	0.378	apoC-I	1.832	±	1.124
ApoD	0.956	±	0.339	apoA-IV	0.950	±	1.407
ApoC-II	0.924	±	0.187	α-2-HS-GP	0.836	±	0.370
LCAT	0.269	±	0.168	apoM	0.320	±	0.123
SAA4	0.242	±	0.108	apoC-III	0.310	±	0.113
PON3	0.226	±	0.120	transferrin	0.248	±	0.097
SAA1	0.196	±	0.074	SAA4	0.208	±	0.074
ApoF	0.172	±	0.054	apoD	0.153	±	0.051
ApoL1	0.098	±	0.050	apoE	0.107	±	0.038
ApoC-IV	0.006	±	0.004	hemopexin	0.110	±	0.026
				prothrombin	0.104	±	0.023
				SAA1	0.085	±	0.079
				Ig κ ch. C	0.048	±	0.048
				Ig γ-1 ch. C	0.029	±	0.014
				α-1b-GP	0.023	±	0.013
				Ig λ-1 ch. C	0.007	±	0.006

Results represent 3 independent measurements in duplicate of isolated HDL from pooled human sera. List represents all proteins detected in at least two out of six samples per group. The complete list can be found in Supplemental Table 4. apo. apoprotein; AT. antitrypsin; ch. chain; Compl.. complement; GP. glycoprotein; Ig. immunoglobulin; LCAT. lecithin cholesterylester transfer protein; PON. paraoxonase; SAA. serum amyloid A; UC. ultracentrifugation.

Supplemental Table 3: Summary of study characteristics of recently published articles used to calculate the protein abundance of HDL-associated proteins.				
**Study**	**Isolation technique**	**HDL Type analyzed**	**Mass spectrometry technique**	**Raw data reported**
**Vaisar et al.** 2007, JCI[1]	Sequential density gradient UC	Total HDL	Shotgun LC-ESI	Summed spectral counts of 20 healthy subjects
**Davidson et al.** 2009, ATVB[2]	Sequential density gradient UC	Total HDL	Shotgun LC-ESI	Summed spectral counts of 9 healthy subjects
**Holzer et al.** 2011, JASN[3]	One-step density gradient UC	Total HDL	Shotgun LC-ESI	Mean spectral counts of 19 healthy subjects
**Holzer et al.** 2012, JLR[4]	One-step density gradient UC	Total HDL	Shotgun LC-ESI	Mean spectral counts of 15 healthy subjects
**Weichhart et al.** 2012, JASN[5]	Sequential density gradient UC	Total HDL	Shotgun LC-ESI	Mean spectral counts of 20 healthy subjects
**Sreckovic et al**. 2013, BBA[6]	Sequential density gradient UC	Total HDL	Shotgun LC-ESI	Summed spectral counts of 11 healthy subjects
**Riwanto et al**. 2013, Circulation[7]	Sequential density gradient UC	Total HDL	Shotgun LC-ESI	Median spectral counts of 20 healthy subjects

Supplemental Table 4: Raw data (spectral counts) collected from recently published studies investigating the proteome of HDL								
		Number of spectral counts reported						
Acc #	Protein name	**Vaisar** *2007.JCI*[1]	**Davidson** *2009. ATVB*[2]	**Holzer** *2011. JASN*[3]	**Holzer** *2012. JLR*[4]	**Weichhart** *2012. JASN*[5]	**Sreckovic** *2013. BBA*[6]	**Riwanto** *2013. Circ*[7]
P02647	apolipoprotein A-I	1013	2258	416.2	477.44	375	6014	512.5
P02652	apolipoprotein A-II	342	503	72.3	39.3	75	1474	43
P02768	HSA	689	21	13.5	66.52	222	900	39
P04114	apolipoprotein B	58	833		15.11	13	160	350.5
P02649	apolipoprotein E	180	507	45	24.65	111	246	136.5
P02656	apolipoprotein C-III	105	116	33.5	35.41	43	584	25
P02655	apolipoprotein C-II	101	116	8.5	4.82	10	584	19
P02654	apolipoprotein C-I	74	248	30.2	28.7	38	307	29
P35542	serum amyloid A4	124	326	37.9	17.64	49	120	51.5
P05090	apolipoprotein D	70	219	16.2	10.92	75	88	77
O95445	apolipoprotein M	74	280	11.8	6.65	32	19	30.5
O14791	apolipoprotein L1	63	214	0.2	6.68	34	58	55.5
P27169	paraoxonase 1	119	22	0.4	18.78	62	80	48
P06727	apolipoprotein A-IV	139	9		4.9	125		56.5
P01009	α-1-antitrypsin	191	12		4.26		81	38.5
P02735	SAA 1/2	92	74	5.6	4.55	25	7	11.5
Q13790	apolipoprotein F	40	93		2.12	11		7
P02749	apolipoprotein H	82				3	58	2
P08519	apolipoprotein(a)		47	0.4	2.04	9		45
P10909	apolipoprotein J	48	14		0.68	22		14
P02766	transthyretin	71	3		1.79	19		
P55058	PLTP	28	30			10		19.5
P00739	HRP	39	13			29		4
P02765	α-2-HS-GP	59			1.19	9	7	3.5
P04180	LCAT	61				8		5.5
Q15166	paraoxonase 3	31	17			14		12.5
P02774	vitamin D BP	65			0.5	7	1	
P01024	complement C3	18			3.55	5		34
Q9UHG3	prenylcystein oxidase	34				3		23
P55056	apolipoprotein C-IV		9		1.95		44	4.5
P04004	vitronectin	42				9		1
P0C0L4	complement C4-A	40				6		
P02787	serotransferrin	10			2.11	23	7	2
P02748	complement C9	30						5.5
P02671	fibrinogen α chain	32	3					
Q14624	α trypsin inhibitor 4	31						1
P00738	haptoglobin				0.91	25		2
P02775	platelet basic protein		14		4.08	5		
P01857	Ig γ-1 chain C region				3.48	13	3	
P02790	hemopexin	15			0.87	3		
P36955	PEDF	15				3		
P01042	kininogen-1	15						
P68871	hemoglobin subunit β					12		1.5
P02763	α-1-acid GP 1				0.89	12		
P0C0L5	complement C4-B				0.88			11.5
P43034	PAF-AH		11					1
P00734	prothrombin		7		3.52			
P04217	α-1B-glycoprotein					10		
P69905	hemoglobin subunit α					3	4	2
P19652	α-1-acid GP 2	1				4		3
P58335	anthrax toxin R2							8
P81605	dermcidin							7.5
P80108	GPLD1							7.5

P01008	antithrombin-III					4		2
P25311	zinc-α-2-glycoprotein					6		
P04003	C4b-binding protein							5
P00450	ceruloplasmin					5		
Q96QR1	SCGB3A1					5		
Q6GMX6	IGH@ protein							4.5
P08514	integrin α-IIb							4.5
P01834	Ig kappa chain C				3.49			1
P02276	platelet factor 4			0.6	3.48			
P23280	carbonic anhydrase 6							4
P49913	CAMP							4
P41240	CSK					4		
P05106	integrin β-3							3.5
P01019	angiotensinogen							3
Q9NR31	SAR1a							3
Q86YZ3	hornerin							3
P14923	junction plakoglobin							3
P18428	LPS binding protein							3
Q9H8L6	multimerin-2							3
Q15465	sonic hedgehog							3
Q9HDC9	APMAP							2.5
P15144	aminopeptidase N							2.5
P15924	desmoplakin							2.5
Q9HCU0	endosialin							2.5
Q14149	MORC3							2.5
P05155	PPC1 inhibitor							2.5
P02760	protein AMBP				0.48	1		1
P01011	α-1-antichymotrypsin							2
P08697	α-2-antiplasmin							2
Q9H6X2	anthrax toxin R1							2
Q9NX62	Inositolphosphatase 3							2
P17301	integrin α-2							2
P05556	integrin β-1							2
P07988	surfactant protein B							2
Q12907	VIP36							2
P01023	α-2-macroglobulin							1.5
Q6Q788	apolipoprotein A-V							1.5
P05546	heparin cofactor 2							1.5
Q29946	HLA-A protein							1.5
P11597	CETP							1
P00746	complement factor D					1		
P26232	catenin α-2			0.6				
P02753	RBP4				0.3			
P01876	Ig α-1 chain C				0.12			
P0CG05	Ig λ-2 chain C				0.11			
**Sum spectral counts**		**4241**	**6019**	**692.9**	**804.87**	**1592**	**10846**	**1840**

APMAP, adipocyte plasma membrane-associated protein; BP, binding protein; CAMP, cathelicidin antimicrobial peptide; CETP, cholesteryl ester transfer protein; CSK, tyrosine-protein kinase c-src; GP, glycoprotein; GPLD1, Glycoprotein phospholipase D, member 1; HRP, haptoglobin related protein; HSA, human serum albumin; LCAT, lecithin:cholesterol acyltransferase; LPS, lipopolysaccharide; MORC3, MORC family CW-type zinc finger protein 3; PEDF, pigment epithelium-derived factor; PAF-AH, platelet activating factor aryl hydrolase; PLTP; phospholipid transfer protein; PPC1, plasma protease C1; R, receptor; RPB4, retinol binding protein 4; SAA, serum amyloid A; SAR1A, GTP-binding protein SAR1a; SCGB3A1, secretoglobin family 3A member 1; VIP, vesicular integral-membrane protein;

Supplemental Table 5: Semi-quantitative estimates of protein abundance based on recently published research papers describing the HDL proteome

		% of total spectral counts							
**Acc. #**	**Protein name**	**Vaisar** *2007.JCI*[1]	**Davidson** *2009. ATVB*[2]	**Holzer** *2011. JASN*[3]	**Holzer** *2012. JLR*[4]	**Weichhart** *2012. JASN*[5]	**Sreckovic** *2013. BBA*[6]	**Riwanto** *2013. Circ*[7]	**Average (%)**
P02647	apolipoprotein A-I	23.886	37.515	60.066	59.319	23.555	55.449	27.853	**41.092**
P02652	apolipoprotein A-II	8.064	8.357	10.434	4.883	4.711	13.590	2.337	**7.482**
P02768	HSA	16.246	0.349	1.948	8.265	13.945	8.298	2.120	**7.310**
P02649	apolipoprotein E	4.244	8.423	6.494	3.063	6.972	2.268	7.418	**5.555**
P04114	apolipoprotein B	1.368	13.840	-	1.877	0.817	1.475	19.049	**5.489**
P02656	apolipoprotein C-III	2.476	1.927	4.835	4.399	2.701	5.384	1.359	**3.297**
P35542	serum amyloid A4	2.924	5.416	5.470	2.192	3.078	1.106	2.799	**3.284**
P02654	apolipoprotein C-I	1.745	4.120	4.358	3.566	2.387	2.831	1.576	**2.940**
P05090	apolipoprotein D	1.651	3.638	2.338	1.357	4.711	0.811	4.185	**2.670**
P06727	apolipoprotein A-IV	3.278	0.150	-	0.609	7.852	-	3.071	**2.137**
P02655	apolipoprotein C-II	2.382	1.927	1.227	0.599	0.628	5.384	1.033	**1.883**
P27169	paraoxonase 1	2.806	0.366	0.058	2.333	3.894	0.738	2.609	**1.829**
O95445	apolipoprotein M	1.745	4.652	1.703	0.826	2.010	0.175	1.658	**1.824**
O14791	apolipoprotein L1	1.485	3.555	0.029	0.830	2.136	0.535	3.016	**1.655**
P01009	α-1-antitrypsin	4.504	0.199	-	0.529	-	0.747	2.092	**1.153**
P02735	SAA 1/2	2.169	1.229	0.808	0.565	1.570	0.065	0.625	**1.005**
P08519	apolipoprotein(a)	-	0.781	0.058	0.253	0.565	-	2.446	**0.586**
Q13790	apolipoprotein F	0.943	1.545	-	0.263	0.691	-	0.380	**0.546**
P10909	apolipoprotein J	1.132	0.233	-	0.084	1.382	-	0.761	**0.513**
P00739	HRP	0.920	0.216	-	-	1.822	-	0.217	**0.454**
P02766	transthyretin	1.674	0.050	-	0.222	1.193	-	-	**0.449**
P01024	complement C3	0.424	-	-	0.441	0.314	-	1.848	**0.432**
P55058	PLTP	0.660	0.498	-	-	0.628	-	1.060	**0.407**
P02749	apolipoprotein H	1.934	-	-	-	0.188	0.535	0.109	**0.395**
Q15166	paraoxonase 3	0.731	0.282	-	-	0.879	-	0.679	**0.367**
P02765	α-2-HS-GP	1.391	-	-	0.148	0.565	0.065	0.190	**0.337**
Q9UHG3	prenylcystein oxidase	0.802	-	-	-	0.188	-	1.250	**0.320**
P04180	LCAT	1.438	-	-	-	0.503	-	0.299	**0.320**
P02787	serotransferrin	0.236	-	-	0.262	1.445	0.065	0.109	**0.302**
P02774	vitamin D BP	1.533	-	-	0.062	0.440	0.009	-	**0.292**
P00738	haptoglobin	-	-	-	0.113	1.570	-	0.109	**0.256**
P04004	vitronectin	0.990	-	-	-	0.565	-	0.054	**0.230**
P0C0L4	complement C4-A	0.943	-	-	-	0.377	-	-	**0.189**
P01857	Ig γ-1 chain C	-	-	-	0.432	0.817	0.028	-	**0.182**
P02775	platelet basic protein	-	0.233	-	0.507	0.314	-	-	**0.151**
P55056	apolipoprotein C-IV	-	0.150	-	0.242	-	0.406	0.245	**0.149**
P02748	complement C9	0.707	-	-	-	-	-	0.299	**0.144**
P02763	α-1-acid GP 1	-	-	-	0.111	0.754	-	-	**0.123**
P68871	hemoglobin subunit β	-	-	-	-	0.754	-	0.082	**0.119**
P02671	fibrinogen α chain	0.755	0.050	-	-	-	-	-	**0.115**
Q14624	α trypsin inhibitor 4	0.731	-	-	-	-	-	0.054	**0.112**
P0C0L5	complement C4-B	-	-	-	0.109	-	-	0.625	**0.105**
P02790	hemopexin	0.354	-	-	0.108	0.188	-	-	**0.093**
P04217	α-1B-glycoprotein	-	-	-	-	0.628	-	-	**0.090**
P00734	prothrombin	-	0.116	-	0.437	-	-	-	**0.079**
P36955	PEDF	0.354	-	-	-	0.188	-	-	**0.077**
P02276	platelet factor 4	-	-	0.087	0.432	-	-	-	**0.074**
P01834	Ig kappa chain C	-	-	-	0.434	-	-	0.054	**0.070**
P19652	α-1-acid GP 2	0.024	-	-	-	0.251	-	0.163	**0.063**
P58335	anthrax toxin R 2	-	-	-	-	-	-	0.435	**0.062**
P81605	dermcidin	-	-	-	-	-	-	0.408	**0.058**
P80108	GPLD1	-	-	-	-	-	-	0.408	**0.058**

P25311	zinc-α-2-glycoprotein	-	-	-	-	0.377	-	-	**0.054**
P01008	antithrombin-III	-	-	-	-	0.251	-	0.109	**0.051**
P01042	kininogen-1	0.354	-	-	-	-	-	-	**0.051**
P69905	hemoglobin subunit α	-	-	-	-	0.188	0.037	0.109	**0.048**
P00450	ceruloplasmin	-	-	-	-	0.314	-	-	**0.045**
Q96QR1	SCGB3A1	-	-	-	-	0.314	-	-	**0.045**
P04003	C4b-binding protein	-	-	-	-	-	-	0.272	**0.039**
P41240	CSK	-	-	-	-	0.251	-	-	**0.036**
Q6GMX6	IGH@ protein	-	-	-	-	-	-	0.245	**0.035**
P08514	integrin α-IIb	-	-	-	-	-	-	0.245	**0.035**
P43034	PAF-AH	-	0.183	-	-	-	-	0.054	**0.034**
P23280	carbonic anhydrase 6	-	-	-	-	-	-	0.217	**0.031**
P49913	CAMP	-	-	-	-	-	-	0.217	**0.031**
P05106	integrin β-3	-	-	-	-	-	-	0.190	**0.027**
P02760	protein AMBP	-	-	-	0.060	0.063	-	0.054	**0.025**
P01019	angiotensinogen	-	-	-	-	-	-	0.163	**0.023**
Q9NR31	SAR1a	-	-	-	-	-	-	0.163	**0.023**
Q86YZ3	hornerin	-	-	-	-	-	-	0.163	**0.023**
P14923	junction plakoglobin	-	-	-	-	-	-	0.163	**0.023**
P18428	LPS binding protein	-	-	-	-	-	-	0.163	**0.023**
Q9H8L6	multimerin-2	-	-	-	-	-	-	0.163	**0.023**
Q15465	sonic hedgehog	-	-	-	-	-	-	0.163	**0.023**
Q9HDC9	APMAP	-	-	-	-	-	-	0.136	**0.019**
P15144	aminopeptidase N	-	-	-	-	-	-	0.136	**0.019**
P15924	desmoplakin	-	-	-	-	-	-	0.136	**0.019**
Q9HCU0	endosialin	-	-	-	-	-	-	0.136	**0.019**
Q14149	MORC3	-	-	-	-	-	-	0.136	**0.019**
P05155	PPC1 inhibitor	-	-	-	-	-	-	0.136	**0.019**
P01011	α-1-antichymotrypsin	-	-	-	-	-	-	0.109	**0.016**
P08697	α-2-antiplasmin	-	-	-	-	-	-	0.109	**0.016**
Q9H6X2	anthrax toxin R 1	-	-	-	-	-	-	0.109	**0.016**
Q9NX62	inositolphosphatase 3	-	-	-	-	-	-	0.109	**0.016**
P17301	integrin α-2	-	-	-	-	-	-	0.109	**0.016**
P05556	integrin β-1	-	-	-	-	-	-	0.109	**0.016**
P07988	surfactant protein B	-	-	-	-	-	-	0.109	**0.016**
Q12907	VIP36	-	-	-	-	-	-	0.109	**0.016**
P26232	catenin α-2	-	-	0.087	-	-	-	-	**0.012**
P01023	α-2-macroglobulin	-	-	-	-	-	-	0.082	**0.012**
Q6Q788	apolipoprotein A-V	-	-	-	-	-	-	0.082	**0.012**
P05546	heparin cofactor 2	-	-	-	-	-	-	0.082	**0.012**
Q29946	HLA-A protein	-	-	-	-	-	-	0.082	**0.012**
P00746	complement factor D	-	-	-	-	0.063	-	-	**0.009**
P11597	CETP	-	-	-	-	-	-	0.054	**0.008**
P02753	RPB 4	-	-	-	0.037	-	-	-	**0.005**
P01876	Ig α-1 chain C	-	-	-	0.015	-	-	-	**0.002**
P0CG05	Ig λ-2 chain C	-	-	-	0.014	-	-	-	**0.002**
**Sum (%)**		**100**	**100**	**100**	**100**	**100**	**100**	**100**	**100**

APMAP, adipocyte plasma membrane-associated protein; BP, binding protein; CAMP, cathelicidin antimicrobial peptide; CETP, cholesteryl ester transfer protein; CSK, tyrosine-protein kinase c-src; GP, glycoprotein; GPLD1, Glycoprotein phospholipase D, member 1; HRP, haptoglobin related protein; HSA, human serum albumin; LCAT, lecithin:cholesterol acyltransferase; LPS, lipopolysaccharide; MORC3, MORC family CW-type zinc finger protein 3; PEDF, pigment epithelium-derived factor; PAF-AH, platelet activating factor aryl hydrolase; PLTP; phospholipid transfer protein; PPC1, plasma protease C1; R, receptor; RPB4, retinol binding protein 4; SAA, serum amyloid A; SAR1A, GTP-binding protein SAR1a; SCGB3A1, secretoglobin family 3A member 1; VIP, vesicular integral-membrane protein.

Supplemental Table 6a:
Mass spectrometry raw data

							**UC**						**UC-HDL purified by size exclusion chromatography**											
							**HDL**						**$HDL_{2/3}$**						**pre-β HDL containing fraction**					
							1	2	3	4	5	6	1	2	3	4	5	6	1	2	3	4	5	6
code	name	Σ Coverage (%)	Σ# Proteins	Σ#Unique Peptides	Σ# Peptides	Σ# PSMs	Area	Area	Area	Area	Area	Area	Area	Area	Area	Area	Area	Area	Area	Area	Area	Area	Area	Area
G3QY98	apoA-I	87.27	1	81	81	58597	4.5E8	6.6E8	9.3E8	3.4E9	1.2E9	1.8E9	1.6E9	1.2E9	2.E9	1.7E9	1.9E9	7.6E8	9.3E8	1.1E9	9.9E8	1.0E9	7.3E8	1.8E9
P02768	HSA	95.73	1	110	110	12814	3.1E7	3.6E7	7.5E7	2.8E8	7.2E7	1.6E8	-	-	-	-	-	-	2.1E8	2.6E8	4.7E8	2.9E8	1.9E8	3.5E8
P0DJG2	apoA-II	78.00	1	19	19	9156	3.0E8	1.3E8	4.0E8	5.2E8	3.7E8	3.1E8	2.7E8	2.7E8	4.4E8	5.8E8	4.4E8	3.1E8	1.4E8	4.5E7	8.3E7	4.7E7	1.1E8	2.0E8
P02649	apoE	85.17	1	53	53	5095	1.1E7	1.7E7	4.5E7	4.7E7	5.5E7	5.6E7	3.5E7	3.9E7	5.6E7	4.4E7	6.2E7	2.8E7	1.8E6	1.3E6	3.3E6	2.6E6	2.6E6	2.3E6
P27169	PON1	71.55	1	29	31	3664	9.8E6	3.5E6	8.3E6	5.0E7	7.1E6	3.3E7	2.2E7	3.4E7	1.4E7	3.2E7	1.8E7	8.0E6	7.7E7	1.0E8	1.8E8	9.5E7	2.1E8	3.5E8
P05090	apoD	42.33	1	17	17	2671	2.1E6	2.5E6	1.7E7	5.3E7	1.9E7	3.5E7	9.9E6	2.1E7	3.3E7	4.2E7	2.4E7	3.9E6	1.4E6	1.5E6	3.5E6	3.3E6	4.5E6	5.9E6
P02656	apoC-III	62.63	1	8	8	2288	5.7E7	1.5E7	2.4E8	3.0E8	2.7E8	2.5E8	5.5E7	4.8E7	1.E8	1.2E8	5.4E7	5.5E6	6.8E6	6.1E6	5.7E6	9.7E6	6.8E6	3.3E6
P35542	SAA4	60.77	1	12	12	2137	1.6E6	1.3E6	5.2E6	5.8E6	3.5E6	6.0E6	1.2E6	6.4E6	1.1E7	9.3E6	3.7E6	1.4E6	5.6E6	2.4E6	6.5E6	4.1E6	3.2E6	3.5E6
P02654	apoC-I	53.01	1	14	14	1797	3.8E7	3.4E7	5.9E7	5.7E7	3.2E7	3.4E7	5.9E7	1.5E7	6.5E7	2.8E7	6.7E7	6.2E6	5.3E7	1.2E7	5.7E7	5.6E7	2.1E7	2.5E7
P02655	apoC-II	59.41	1	11	11	1639	5.8E6	8.8E6	3.7E7	2.5E7	1.4E7	2.2E7	2.6E7	9.8E6	3.0E7	2.7E7	2.6E7	7.2E6	5.6E7	5.7E7	1.3E8	1.4E8	7.1E7	9.6E7
O95445	apoM	36.17	1	15	15	1256	1.8E6	2.1E6	2.2E7	7.4E7	2.8E7	4.1E7	3.5E7	6.2E7	3.1E7	3.2E7	4.3E7	9.4E5	4.2E6	6.0E6	5.0E6	1.4E7	4.0E6	1.0E7
P06727	apoA-IV	58.84	1	30	30	865	1.4E7	5.2E6	1.6E7	8.3E6	1.1E7	9.3E6	-	-	4.6E6	-	-	-	5.8E7	1.5E6	1.1E7	1.3E7	1.8E6	1.9E7
P01009	a-1-AT	33.97	1	14	14	754	1.4E6	1.7E6	7.8E6	1.1E7	7.2E6	1.5E7	-	4.8E5	-	-	-	-	6.0E7	3.3E7	1.1E8	1.4E8	6.1E7	9.3E7
P02765	a-2-HS-GP	43.32	1	16	16	580	8.5E5	5.0E5	1.7E6	5.0E6	3.4E6	2.9E6	-	-	-	-	-	-	1.3E7	1.7E7	3.1E7	2.3E7	9.9E6	1.2E7
O14791	apoL1	41.46	1	18	18	518	1.0E6	4.9E5	4.0E5	2.2E6	1.6E6	6.8E5	3.3E6	2.1E6	5.4E6	1.4E6	8.9E5	2.7E5	-	-	-	-	-	-
P0DJI8	SAA1	70.49	1	6	6	211	-	1.2E6	-	2.8E6	6.1E6	6.3E6	3.2E5	3.0E6	5.8E6	8.2E6	4.7E6	3.3E6	-	-	1.4E6	7.3E6	3.3E5	3.8E6
P10909	Clusterin	21.83	1	9	9	193	-	2.7E5	-	-	-	-	-	-	-	-	-	-	-	-	-	-	-	-
P00734	Protrombin	8.68	1	3	3	192	1.3E6	1.6E6	1.1E6	9.9E6	6.2E6	4.2E6	-	-	-	-	-	-	1.8E6	1.4E6	2.0E6	3.3E6	2.6E6	1.9E6
P01024	Compl. C3	14.67	1	16	16	122	7.6E4	3.3E5	-	-	-	-	-	7.0E5	-	-	-	-	-	-	-	-	-	-
P02787	TF	28.22	1	14	14	121	-	-	-	-	-	-	-	-	-	-	-	-	1.5E6	4.1E6	6.5E6	9.3E6	4.4E6	8.0E6
P04180	LCAT	20.23	1	5	5	119	9.2E5	1.2E6	3.3E6	1.8E7	1.8E6	-	6.2E6	9.1E6	-	1.4E7	-	3.0E6	-	-	-	-	-	-
Q15166	PON3	9.04	1	1	3	62	5.0E5	2.8E5	5.0E6	1.3E7	3.8E6	1.1E7	3.7E6	8.6E6	3.3E6	8.6E6	5.6E6	2.8E5	-	-	-	-	-	1.2E6
P55058	PLTP	8.32	1	6	6	43	-	-	-	-	-	-	-	-	-	-	-	4.7E4	-	-	-	-	-	-
P01876	Ig a-1	20.96	1	6	6	40	-	-	-	-	-	-	-	1.1E6	-	-	-	-	-	-	-	-	-	-
P05534	HLA-A	25.75	2	6	6	38	-	-	-	-	-	-	4.7E5	-	-	-	-	-	-	-	-	-	-	-
P01857	Ig g-1	20.30	1	5	5	36	2.6E5	1.9E5	-	8.2E5	-	-	5.5E5	-	-	-	-	-	1.4E5	-	8.3E5	1.2E6	7.5E5	9.4E5
P02790	Hemopexin	14.50	1	4	4	34	-	-	-	-	-	-	-	-	-	-	-	-	7.2E5	3.2E6	2.4E6	6.9E5	3.7E6	3.8E6
P01834	Ig k	70.75	1	4	4	31	-	3.0E5	2.2E6	-	-	-	-	-	-	-	-	-	-	-	6.1E5	-	3.0E6	2.5E6
P04217	a-1B-GP	14.75	1	5	5	20	-	-	-	-	-	-	-	-	-	-	-	-	-	-	7.6E5	9.5E5	5.9E5	8.4E5
P55056	apoC-IV	21.26	1	3	3	15	-	1.2E5	2.0E5	1.8E5	7.4E4	-	2.4E5	2.8E4	3.7E5	-	2.7E5	-	-	-	-	-	-	-
Q13790	apoF	8.28	1	2	2	14	4.3E5	9.7E5	1.9E6	8.0E6	1.5E6	9.6E6	2.4E6	5.2E6	3.4E6	4.6E6	5.6E6	1.2E6	-	-	-	-	-	-
P08519	apo(a)	16.71	1	2	2	5	-	-	-	-	-	-	-	-	-	-	5.1E5	-	-	-	-	-	-	-
P0CG04	Ig l-1	21.70	4	2	2	3	-	-	1.8E5	-	6.6E5	-	-	9.2E5	-	-	-	-	-	-	1.7E5	-	3.8E5	3.8E5
P81605	Dermcidin	17.27	1	2	2	2	-	-	-	-	-	-	-	-	-	-	-	-	-	-	-	-	-	-
AREA SUM							9.3E8	9.3E8	1.9E9	4.8E9	2.1E9	2.8E9	2.1E9	1.7E9	2.8E9	2.7E9	2.7E9	1.1E9	1.6E9	1.6E9	2.1E9	1.9E9	1.4E9	3.0E9

Raw data from individual mass spectrometry measurements are shown. Data on the left indicate the coverage of the protein sequence (Σ Coverage), the number of identified proteins (Σ#Proteins), the number of unique peptides (Σ Unique Peptides), the number of total peptides (Σ#Peptides) and the total number of identified peptide spectra matched for the protein (PSMs) for all 54 samples analyzed. Raw data for individual measurements are represented as areas under curve (Area) and were used for quantification. "-", not detected; apo, apoprotein; AT, antitrypsin; BP, binding protein; Compl., complement; GP, glycoprotein; HSA, human serum albumin; HRP, haptoglobin related protein; LCAT, lecithin cholesterylester transfer protein; PLTP, phospholipid transfer protein; PON, paraoxonase; SAA, serum amyloid A; TF, transferrin; UC, ultracentrifugation.

Supplemental Table 6b:
MS raw data

		UC-HDL purified by native gel electrophoresis												UC-HDL purified by native gel electrophoresis									
		$HDL_{2/3}$						pre-β HDL containing fraction						HDL_2					HDL_3				
		1	2	3	4	5	6	1	2	3	4	5	6	1	2	3	4	5	1	2	3	4	5
code	name	Area	Area	Area	Area	Area	Area	Area	Area	Area	Area	Area	Area	Area	Area	Area	Area	Area	Area	Area	Area	Area	Area
G3QY98	apoA-I	4.7E8	2.9E8	4.7E8	4.0E8	2.0E8	2.7E8	1.9E7	5.6E6	2.7E6	8.7E6	9.3E6	8.5E6	6.3E7	4.5E7	5.9E7	5.9E7	5.2E7	3.8E8	2.6E8	1.9E8	6.7E7	8.3E7
P02768	HSA	-	-	-	-	-	-	1.6E7	1.6E6	1.7E6	2.9E6	1.9E6	9.0E6	-	-	-	-	-	-	-	-	-	-
P0DJG2	apoA-II	2.1E8	5.9E7	6.2E7	9.1E7	2.2E7	2.9E7	1.1E6	1.3E6	4.2E5	2.0E6	2.8E5	1.2E5	1.4E7	5.1E6	7.7E6	5.2E6	5.4E6	6.5E7	3.7E7	4.8E7	3.6E7	1.4E7
P02649	apoE	1.4E7	6.8E6	1.1E7	7.8E6	2.8E6	4.7E6	-	-	-	-	-	-	4.5E5	2.2E6	1.9E6	3.6E6	2.5E6	9.7E5	3.1E5	-	-	3.2E5
P27169	PON1	2.1E7	1.3E7	8.0E6	4.0E6	3.9E6	2.3E6	3.4E6	8.0E5	1.5E6	2.5E6	1.5E6	8.6E6	1.0E6	1.4E6	-	-	-	1.5E7	2.9E6	1.7E6	4.3E6	8.6E5
P05090	apoD	2.5E6	6.3E5	1.7E6	7.6E5	2.9E5	4.2E5	5.6E5	-	-	-	-	-	-	-	-	-	1.3E5	1.1E6	1.1E6	-	-	7.8E5
P02656	apoC-III	3.7E5	4.7E5	2.5E6	2.3E6	5.3E5	6.0E5	2.8E6	2.4E5	6.0E5	2.2E6	1.8E5	8.6E5	-	-	-	2.0E5	-	-	1.1E6	-	-	4.6E5
P35542	SAA4	1.3E7	2.1E6	3.5E6	2.2E6	2.5E5	1.7E6	-	-	-	-	-	-	-	4.2E5	-	3.8E5	1.7E5	3.6E6	2.1E6	2.8E6	1.5E6	5.5E5
P02654	apoC-I	8.4E5	5.7E5	8.6E5	2.6E5	2.7E5	1.4E5	-	-	-	-	-	-	5.1E5	1.9E5	8.7E5	3.9E5	-	7.3E5	-	-	-	-
P02655	apoC-II	-	-	-	-	-	-	-	-	-	-	-	-	-	-	-	-	-	-	-	-	-	-
O95445	apoM	-	-	-	-	-	-	-	-	-	-	-	-	-	-	-	-	-	-	-	-	-	-
P06727	apoA-IV	-	-	-	-	-	-	1.2E6	1.9E5	7.1E5	8.5E5	2.2E5	6.1E5	-	-	-	-	-	-	-	-	-	-
P01009	a-1-AT	-	-	-	-	-	-	1.3E6	4.3E5	6.8E5	1.4E6	1.4E6	1.0E6	-	-	-	-	-	-	-	-	-	-
P02765	a-2-HS-GP	-	-	-	-	-	-	1.0E6	-	-	8.6E4	-	-	-	-	-	-	-	-	-	-	-	-
O14791	apoL1	2.9E6	1.6E6	1.4E6	3.5E5	-	-	-	-	-	-	-	-	-	2.3E5	-	2.7E5	-	1.1E6	2.1E5	-	-	-
P0DJI8	SAA1	-	-	-	-	-	-	-	-	-	-	-	-	-	-	-	-	-	-	-	-	-	-
P10909	Clusterin	-	-	-	-	-	-	-	-	-	-	-	-	-	2.5E5	-	-	-	-	-	-	-	-
P00734	thrombin	-	-	-	-	-	-	-	-	-	-	-	-	-	-	-	-	-	-	-	-	-	-
P01024	Compl. C3	-	-	-	-	-	-	-	-	-	-	-	-	-	-	-	-	-	-	-	-	-	-
P02787	TF	-	-	-	-	-	-	-	-	-	-	-	-	-	-	-	-	-	-	-	-	-	-
P04180	LCAT	-	-	-	-	-	-	3.0E5	4.2E5	3.5E5	8.0E5	6.3E5	-	-	-	-	-	-	-	-	-	-	-
Q15166	PON3	-	-	-	-	-	-	-	3.3E4	-	-	8.7E4	-	-	-	-	-	-	4.2E6	-	-	-	-
P55058	PLTP	-	-	-	-	-	-	-	-	-	-	-	-	-	7.2E4	-	-	-	-	-	-	-	-
P01876	Ig a-1	-	-	-	-	-	-	-	-	-	-	-	-	-	-	-	-	-	-	-	-	-	-
P05534	HLA-A	-	-	-	-	-	-	-	-	-	-	-	-	-	-	-	-	-	-	-	-	-	-
P01857	Ig g-1	-	-	-	-	-	-	-	-	-	-	-	-	-	-	-	-	-	-	-	-	-	-
P02790	hemopexin	-	-	-	-	-	-	-	-	-	-	-	-	-	-	-	-	-	-	-	-	-	-
P01834	Ig k	-	-	-	-	-	-	-	-	-	-	-	-	-	-	-	-	-	-	-	-	-	-
P04217	a-1B-GP	-	-	-	-	-	-	-	-	-	-	-	-	-	-	-	-	-	-	-	-	-	-
P55056	apoC-IV	-	-	-	-	-	-	-	-	-	-	-	-	-	-	-	-	-	-	-	-	-	-
Q13790	apoF	2.9E4	4.0E5	5.7E5	-	-	-	-	-	-	-	-	-	3.2E5	-	-	-	-	-	-	-	-	-
P08519	apo(a)	-	-	-	-	-	-	-	-	-	-	-	-	-	-	-	1.1E5	-	-	-	-	-	-
P0CG04	Ig l-1	-	-	-	-	-	-	-	-	-	-	-	-	-	-	-	-	-	-	-	-	-	-
P81605	Dermcidin	-	-	-	-	-	-	4.7E5	-	-	1.0E5	-	-	-	-	-	-	-	-	-	7.5E4	-	-
AREA SUM		7.3E8	3.8E8	5.6E8	5.1E8	2.3E8	3.1E8	4.8E7	1.1E7	8.6E6	2.1E7	1.6E7	2.9E7	7.9E7	5.5E7	7.0E7	6.9E7	6.1E7	4.7E8	3.1E8	2.4E8	1.1E8	1.0E8

Raw data from individual mass spectrometry measurements are shown. Data on the left indicate the coverage of the protein sequence (Σ Coverage), the number of identified proteins (Σ#Proteins), the number of unique peptides (Σ Unique Peptides), the number of total peptides (Σ#Peptides) and the total number of identified peptide spectra matched for the protein (PSMs) for all 54 samples analyzed. Raw data for individual measurements are represented as areas under curve (Area) and were used for quantification. “-“, not detected; apo, apoprotein; AT, antitrypsin; BP, binding protein; Compl., complement; GP, glycoprotein; HSA, human serum albumin; HRP, haptoglobin related protein; LCAT, lecithin cholesterylester transfer protein; PLTP, phospholipid transfer protein; PON, paraoxonase; SAA, serum amyloid A; TF, transferrin; UC, ultracentrifugation.

Supplemental References:

1. Vaisar, T. *et al*. Shotgun proteomics implicates protease inhibition and complement activation in the antiinflammatory properties of HDL. *J. Clin. Invest.* **117**, 746-756 (2007).

2. Davidson, W. S. *et al*. Proteomic analysis of defined HDL subpopulations reveals particle-specific protein clusters: relevance to antioxidative function. *Arterioscler. Thromb. Vasc. Biol.* **29**, 870-876 (2009).

3. Holzer, M. *et al*. Uremia alters HDL composition and function. *J. Am. Soc. Nephrol.* **22**, 1631-1641 (2011).

4. Holzer, M. *et al*. Psoriasis alters HDL composition and cholesterol efflux capacity. *J. Lipid Res.* **53**, 1618-1624 (2012).

5. Weichhart, T. *et al*. Serum amyloid A in uremic HDL promotes inflammation. *J. Am. Soc. Nephrol.* **23**, 934-947 (2012).

6. Sreckovic, I. *et al*. Distinct composition of human fetal HDL attenuates its anti-oxidative capacity. *Biochim. Biophys. Acta* **1831**, 737-746 (2013).

7. Riwanto, M. *et al*. Altered activation of endothelial anti- and proapoptotic pathways by high-density lipoprotein from patients with coronary artery disease: role of high-density lipoprotein-proteome remodeling. *Circulation* **127**, 891-904 (2013).
